# Periodontitis and early mortality among adults treated with hemodialysis: a multinational propensity-matched cohort study

**DOI:** 10.1186/s12882-017-0574-x

**Published:** 2017-05-22

**Authors:** Marinella Ruospo, Suetonia C Palmer, Germaine Wong, Jonathan C Craig, Massimo Petruzzi, Michele De Benedittis, Pauline Ford, David W Johnson, Marcello Tonelli, Patrizia Natale, Valeria Saglimbene, Fabio Pellegrini, Eduardo Celia, Ruben Gelfman, Miguel R Leal, Marietta Torok, Paul Stroumza, Anna Bednarek-Skublewska, Jan Dulawa, Luc Frantzen, Domingo del Castillo, Staffan Schon, Amparo G Bernat, Jorgen Hegbrant, Charlotta Wollheim, Letizia Gargano, Casper P. Bots, Giovanni FM Strippoli, on behalf of the ORALD Investigators

**Affiliations:** 1 Diaverum Medical Scientific Office, Lund, Sweden; 2 Amedeo Avogadro University of Eastern Piedmont, Novara, Italy; 3 University of Otago Christchurch, Christchurch, New Zealand; 4 University of Sydney, Sydney, Australia; 5 Department of Emergency and Organ Transplantation, University of Bari, Piazza Giulio Cesare, 70124 Bari, Italy; 6 University of Queensland, Brisbane, Australia; 7 Translational Research Institute, Brisbane, Australia; 8 University of Calgary, Calgary, Canada; 9 Global Medical Biogen Idec, Cambridge, MA USA; 10 Medical University of Lublin, Lublin, Poland; 11 SHS, Medical University of Silesia, Katowice, Poland; 12 Academic Centre for Dentistry Amsterdam (ACTA), Amsterdam, The Netherlands; 13 Diaverum Academy, Lund, Sweden

**Keywords:** End-stage kidney disease, Hemodialysis, Oral health, Periodontal disease, All-cause mortality

## Abstract

**Background:**

Periodontitis is associated with cardiovascular mortality in the general population and adults with chronic diseases. However, it is unclear whether periodontitis predicts survival in the setting of kidney failure.

**Methods:**

ORAL-D was a propensity matched analysis in 3338 dentate adults with end-stage kidney disease treated in a hemodialysis network in Europe and South America designed to examine the association between periodontitis and all-cause and cardiovascular-related mortality in people on long-term hemodialysis. Participants were matched 1:1 on their propensity score for moderate to severe periodontitis assessed using the World Health Organization Community Periodontal Index.

A random-effects Cox proportional hazards model was fitted with shared frailty to account for clustering of mortality risk within countries.

**Results:**

Among the 3338 dentate participants, 1355 (40.6%) had moderate to severe periodontitis at baseline. After using propensity score methods to generate a matched cohort of participants with periodontitis similar to those with none or mild periodontal disease, moderate to severe periodontitis was associated with a lower risk of all-cause (9.1 versus 13.0 per 100 person years, hazard ratio 0.74, 95% confidence interval 0.61 to 0.90) and cardiovascular (4.3 versus 6.9 per 100 person years, hazard ratio 0.67, 0.51 to 0.88) mortality. These associations were not changed substantially when participants were limited to those with 12 or more natural teeth and when accounting for competing causes of cardiovascular death.

**Conclusion:**

In contrast to the general population, periodontitis does not appear to be associated with an increased risk of early death in adults treated with hemodialysis.

**Electronic supplementary material:**

The online version of this article (doi:10.1186/s12882-017-0574-x) contains supplementary material, which is available to authorized users.

## Background

Adults with end-stage kidney disease experience a 10 to 20% annual risk of mortality [1] and have severe symptoms including fatigue, anorexia, itch, and sleep disturbance. Despite decades of clinical trials, specific interventions including statin therapy, antiplatelet agents, higher dialysis dose, early dialysis start and cinacalcet have generally not been shown to improve mortality in this clinical setting, [2–5] and new modifiable health determinants need to be identified. Evaluation of candidate interventions in people on dialysis is challenging due to reverse epidemiology, in which risk factors for poorer health in populations without advanced kidney disease, such as high body mass index, [6] cholesterol, [7] and blood pressure, [8] are paradoxically associated with better survival among adults with end-stage kidney disease.

Periodontitis, a multifactorial disease that involves the structures supporting the teeth, is common, treatable, and may be associated with mortality in the general population. [9, 10] Approximately 10% of adults with chronic kidney disease have evidence of moderate to severe periodontitis. [11] Periodontitis is associated with systemic inflammation [12, 13], which is implicated in the pathogenesis of cardiovascular disease caused by kidney failure [14] and such inflammation is consistently associated with worse survival in adults undergoing long-term hemodialysis [15, 16]. In our recent systematic review of dental disease in adults with chronic kidney disease [17], we identified two cohort studies that showed increased mortality among dialysis patients with periodontitis, although these studies had small sample sizes (*n* = 253 and *n* = 168, respectively) and remained at risk of bias from confounding requiring additional confirmation from larger studies in the dialysis population [18, 19]. A recent third study in a large older cohort with a range of kidney disease including those with normal kidney function, periodontitis was associated with premature mortality [20]. And similarly, a study among 1300 adults in the third national health and nutrition examination dataset reported an increased risks of mortality (hazard ratio 1.35) among people with chronic kidney disease and periodontitis compared with those without periodontitis [11]. Randomized studies evaluating the effects of periodontal treatment in people with chronic kidney disease are ongoing, [21–23] although a recent trial assessing non-surgical periodontal therapy in adults with diabetes was discontinued due to futility [24] and a recent exploratory randomized controlled trial of intensive periodontal therapy among 53 dialysis patients did not show sustained effects of periodontal treatment at six months or differences in inflammatory markers during follow-up [25].

The Oral Diseases in Hemodialysis study (ORAL-D) [26] was designed to determine whether oral disease was associated with all-cause and cardiovascular mortality in adults with treated with hemodialysis. In this report, the association of periodontal disease with mortality outcomes from the ORAL-D study is evaluated in a larger dataset than has been available in previous cohort studies.

## Methods

### Study population

ORAL-D was a multinational, prospective cohort study of oral disease and dental health practices in adults with end-stage kidney disease (Additional file 1) [26]. Adults aged 18 years or older who were treated with long-term hemodialysis for any duration were enrolled between July 2010 and February 2012. Consecutive participants were recruited from clinics within a private dialysis provider network in Argentina, France, Hungary, Italy, Poland, Portugal, and Spain. Participants were excluded if they had cognitive impairment or could not provide informed consent. Ethics approval was obtained from all relevant institutional ethics committees and was conducted in accordance with the Declaration of Helsinki. All participants provided written and informed consent before participation. The study has been reported according to the Strengthening the Reporting of Observational studies in Epidemiology (STROBE) guidelines.

### Measurement of covariates

Demographic, clinical and dialysis-related data were obtained from a centralized database linked to the participants via their unique provider identification code. Standardized variables, including age, gender, race, and country of treatment, education level, marital and occupational status, alcohol intake, smoking history, physical activity, housing situation, family income and financial stress, self-reported comorbidities (including cardiovascular disease and diabetes), body mass index, medication use, serum parameters including hemoglobin, phosphorus, parathyroid hormone, calcium, and albumin, and dialysis characteristics were extracted. All variables were assessed and recorded using the same standard operating procedures across all countries and clinical sites.

### Measurement of exposure

All participants underwent an oral examination with a dentist who was trained in periodontology before the start of an outpatient dialysis treatment. The oral examination was conducted according to a dental practice manual outlining all the procedures to be conducted by dentists within each clinic, which was translated into all languages of the clinical teams. The manual described the full oral examination according to World Health Organization guidelines [27]. All dentists participated in a teleconference to discuss and calibrate their approach to the protocol before examining patients at each study center.

The full mouth periodontal examination, performed with a standardized dental CPI-probe (WHO-probe), included assessments of the periodontal probing depth (PPD), the clinical attachment loss (CAL) score and the bleeding on probing (BOP) indices (described in Additional file 2). Periodontal health was then categorized using the World Health Organization community periodontal index (CPI) which identifies five CPI scores as: normal (CPI 0), gingival bleeding (CPI 1), calculus (CPI 2), shallow periodontal pocket depth of 4–5 mm (CPI 3), and deep periodontal pocket depth of 6 mm of more (CPI 4). The following severities of periodontitis were assigned: none = CPI 0; mild = CPI 1 to CPI 2; moderate = CPI 3, and severe = CPI 4 [28]. Periodontitis was thus defined for the basis of propensity score calculations as: none or mild (corresponding to CPI 0–2) or moderate to severe (CPI 3–4).

### Measurement of outcomes

The outcomes of interest were all-cause mortality and death due to cardiovascular causes. Patients who withdrew from the study, underwent kidney transplantation, were lost to follow up, or survived were censored at the end of the follow up period. Data for total and cause-specific mortality were obtained using data linkage with a centralized database within the dialysis provider network in which changes to patient status were updated on a monthly basis by the managing clinicians who were unaware of periodontitis status. Cardiovascular death was defined as sudden death or death attributed to acute myocardial infarction, pericarditis, atherosclerotic heart disease, cardiomyopathy, cardiac arrhythmia, cardiac arrest, valvular heart disease, pulmonary edema, congestive cardiac failure, cerebrovascular accident (including intracranial hemorrhage), ischemic brain damage (including anoxic encephalopathy), or mesenteric infarction or ischemia of the bowel.

### Statistical analysis

#### Primary analyses

The relevance of periodontitis to the risks of all-cause and cardiovascular mortality was evaluated using multivariable-adjusted, random-effects Cox proportional hazard analyses to take into account the intra-cluster correlation within countries. We used backwards elimination to select variables for inclusion in the multivariate models retaining those (aside from energy intake) which predicted mortality (*p* < 0.05) or with a clinically meaningful impact on the hazard ratio of mortality. The final model included the following variables: age, sex, smoking history (current, former, never), self-reported family income (much higher than average, higher than average, average, lower than average, much lower than average), myocardial infarction, diabetes, body mass index, mean arterial blood pressure, hemoglobin, serum phosphorus, number of teeth, and patient time treated with dialysis (in months).

#### Propensity score methods

Two different propensity score methods, propensity score matching and the inverse probability of treatment weighting (IPTW) using the propensity scores, were used to reduce the effects of confounding when estimating the association of periodontitis with all-cause and cause-specific mortality. To estimate the propensity score, we used logistic regression to obtain the predicted probability of exposure (i.e. the presence and absence of periodontitis). The propensity scores were obtained based upon the most parsimonious and clinically relevant model including the following baseline variables: age, sex, number of teeth, income, smoking, and time treated with dialysis (months). Using this model, we were able to generate propensity score weights for all participants as there were no missing values. Propensity score matching aimed to balance the potential confounding variables of interest between participants with and without periodontitis but not the oral health characteristics indicative of periodontitis. Patients with moderate-severe periodontitis were matched 1-to-1 without replacement using a varying-width caliper-matching algorithm (5-to-1 digit matching). The propensity scores were then checked to ensure they were balanced across the periodontitis and non-periodontitis groups. The balance in covariates was assessed before and after matching using standardized differences; standardized differences of 0.2, 0.5 and 0.8 were considered to represent small, medium and large differences, respectively [29].

The association of periodontitis with mortality outcomes was next estimated using a random-effects Cox proportional hazards model fitted with shared frailty to account for clustering of mortality risk within countries. In the whole cohort, we adjusted the Cox proportional hazards regression for sex, age, number of teeth, income, smoking, income, physical activity, body mass index, myocardial infarction, diabetes, mean arterial pressure, time treated with dialysis, and serum phosphorus. Survival analyses were then conducted solely incorporating a weighting of participant information by their propensity score for moderate to severe periodontitis (inverse probability of treatment weighted (IPTW)) in the random-effects Cox proportional hazards regression model clustered by country. No additional variables were used in the numerator to calculate inverse probability weighting. Finally, survival analyses were conducted using random-effects Cox proportional hazards regression clustered by country in a model that only included the exposure variable denoting periodontitis within the matched cohort.

#### Sensitivity analyses

For the primary propensity score analysis, we used a parsimonious model that could include all 1355 participants with periodontitis (with no missing data) and adjusted for age, sex, number of teeth, income, smoking, and time treated with dialysis. In sensitivity analyses, to check the findings of this parsimonious model, we did additional analyses using propensity score methods, matching all patients (*n* = 1078) based on the full variable set used in the primary regression analysis including age, sex, serum phosphorus, physical activity, body mass index, number of teeth, income, smoking history, myocardial infarction, diabetes, mean arterial pressure, and time on dialysis. This excluded those who had missing data for body mass index and physical activity.

To assess the robustness of the findings and to limit the impact of tooth loss on the results causing misclassification of periodontitis severity as low or absent, analyses were repeated omitting participants with fewer than 12 natural teeth using the propensity matched cohort. Finally, the potential relevance of the competing risk of non-cardiovascular-related death was considered using the Fine and Gray competing risk regression modelling [30] in both the entire and matched cohorts.

All *P* values were two sided and values below 0.05 were considered to be statistically significant for survival analyses. The analyses were performed using SAS 9.3 (www.sas.com).

## Results

**Fig. 1:**
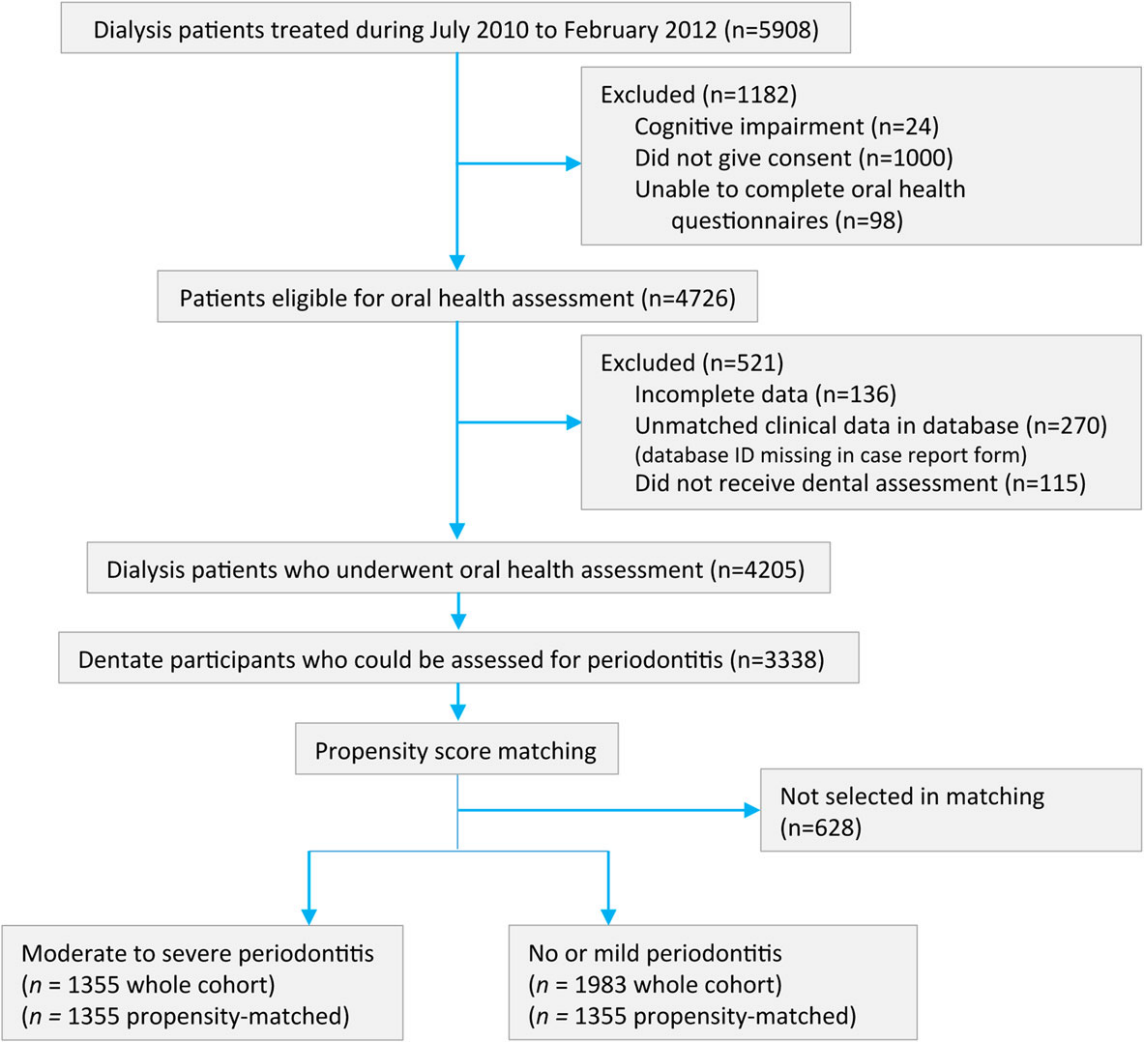
Flow chart of study recruitment and enrolment

Overall, 5908 adults treated with long-term hemodialysis in the dialysis network were assessed for eligibility (Fig. 1). After excluding 1182 who did not provide consent and 521 due to incomplete oral health or clinical data, ORAL-D involved 4205 participants. 3338 (77.2%) participants were dentate and could be assessed for periodontitis, of whom 2229 (66.8%) had 12 natural teeth or more.

Overall, 1355 (40.6%) participants had moderate to severe periodontitis. The clinical and demographic characteristics of participants with moderate to severe periodontitis differed from those with mild or no periodontitis. Participants with moderate or severe periodontitis were older, more likely to be men, were more often married, unemployed or retired, had higher serum albumin levels, and had survived treated with dialysis for longer (Table 1).

**Table 1:** Baseline characteristics of study participants according to periodontal status in unmatched and matched cohorts

Variables	Full cohort			After propensity score matching		
	None or mild periodontitis (*n* = 1983)	Moderate to severe periodontitis (*n* = 1355)	Standardized difference^b^	None or mild periodontitis (*n* = 1355)	Moderate to severe periodontitis (*n* = 1355)	Standardized difference^b^
Demographics						
Age (years)^c^	57.3 (16.3)	61.7 (14.5)	0.23	61.8 (14.5)	61.7 (14.5)	0.005
Country						
Argentina	1304 (65.8)	177 (13.1)	0.99	846 (62.4)	177 (13.1)	0.91
France	28 (1.4)	11 (0.8)	0.05	27 (2.0)	11 (0.8)	0.08
Hungary	182 (9.2)	233 (17.2)	−0.20	141 (10.4)	233 (17.2)	−0.17
Italy	97 (4.9)	312 (23.0)	−0.49	89 (6.6)	312 (23.0)	−0.42
Poland	200 (10.1)	39 (2.9)	0.22	119 (8.8)	39 (2.9)	0.19
Portugal	169 (8.5)	464 (34.2)	−0.58	131 (9.7)	464 (34.2)	−0.55
Spain	3 (0.2)	119 (8.8)	−0.42	2 (0.2)	119 (8.8)	−0.42
Men^c^	1116 (56.3)	852 (62.9)	−0.10	838 (61.9)	852 (62.9)	−0.02
European race	1899 (96.4)	1246 (91.9)	0.17	1297 (95.7)	1246 (91.9)	0.13
Socioeconomic characteristics						
Current or former smoker^c^	381 (31.2)	408 (34.6)	−0.06	352 (26.0)	408 (34.6)	−0.15
Married	859 (60.7)	835 (68.0)	−0.12	583 (61.2)	835 (68.0)	−0.12
Secondary education	466 (39.4)	460 (40.4)	−0.02	331 (38.7)	460 (40.4)	−0.03
Employed	218 (18.1)	148 (12.9)	0.12	149 (16.7)	148 (12.9)	0.09
Family income above domestic average^c^	132 (7.4)	106 (8.2)	−0.02	98 (7.6)	106 (8.2)	−0.02
Body mass index (kg/m^2^)	26.9 (5.2)	26.7 (5.2)	0.04	26.9 (5.2)	26.7 (5.2)	0.04
Comorbid medical conditions						
Myocardial infarction	112 (10.0)	138 (13.3)	−0.09	95 (11.7)	138 (13.3)	−0.04
Stroke	88 (7.8)	125 (12.0)	−0.12	70 (8.6)	125 (12.0)	−0.12
Diabetes mellitus	338 (30.3)	335 (29.4)	0.02	238 (29.7)	335 (29.4)	0.005
Laboratory variables						
Serum albumin (g/dL)	3.7 (0.4)	3.9 (0.4)	−0.41	3.7 (0.4)	3.9 (0.4)	−0.41
Serum phosphorous (mmol/L)	1.6 (0.5)	1.5 (0.5)	0.16	1.5 (0.5)	1.5 (0.5)	0
Serum calcium (mmol/L)	2.2 (0.2)	2.2 (0.2)	0	2.2 (0.2)	8.8 (0.8)	0
Hemoglobin (g/dL)	11.0 (1.4)	11.2 (1.3)	−0.12	11.1 (1.4)	11.2 (1.3)	−0.06
Dialysis characteristics						
Time treated with dialysis (months)^c^	75.1 (54.9)	84.6 (66.4)	−0.13	82.5 (59.1)	84.6 (66.4)	−0.02
Kt/V^a^	1.7 (0.3)	1.7 (0.3)	0	1.6 (0.3)	1.7 (0.3)	−0.27
Mean arterial pressure (mmHg)	89.0 (13.7)	91.7 (13.7)	−0.16	88.8 (13.5)	91.7 (13.7)	−0.17
Oral health practices and dental health						
Number of teeth^c^	16.5 (9.4)	17.1 (8.3)	−0.05	16.5 (9.2)	17.1 (8.3)	−0.05
Number of decayed, missing, filled teeth	19.4 (8.9)	19.2 (8.1)	0.02	19.3 (8.8)	19.2 (8.1)	0.01
Use of dental floss	153 (7.8)	115 (8.6)	−0.02	90 (6.7)	115 (8.6)	−0.06
Brushing teeth twice or more often per day	1310 (67.1)	854 (64.2)	0.05	874 (65.2)	854 (64.2)	0.05

Data are expressed as mean (SD) or number (%). ^a^Kt/V refers to the clearance of urea and is a measure of the amount of dialysis received. Proportions do not always correspond to overall numbers of participants due to missing data. ^b^Standardized differences of 0.2, 0.5 and 0.8 can be considered to represent small, medium and large differences, respectively [29]. These differences do not denote statistical significance. To convert serum phosphorus from mmol/L to mg/dL, divide by 0.323. To convert calcium from mmol/L to mg/dL, multiply by 0.25. ^c^Used in propensity score matching

There were large differences in periodontal characteristics among participants based on their periodontal status. Participants with moderate to severe periodontitis had evidence of deeper periodontal probing depths, more severe clinical attachment loss, increased distance between the cementum-enamel junction and free gingival margin, and more extensive bleeding on probing as expected (Table 2).

**Table 2:** Baseline periodontal characteristics defined by the World Health Organization Community Periodontal Index in unmatched and matched cohorts

Variables	Full cohort			After propensity score matching		
	None or mild periodontitis (*n* = 1983)	Moderate to severe periodontitis (*n* = 1355)	Standardized difference^a^	None or mild periodontitis (*n* = 1355)	Moderate to severe periodontitis (*n* = 1355)	Standardized difference^a^
Periodontal probing depth, mm	0.68 (0.38)	1.53 (0.70)	−1.36	0.70 (0.38)	1.53 (0.70)	−1.33
Clinical attachment loss, mm	2.03 (1.56)	3.15 (1.55)	−0.59	2.12 (1.55)	3.15 (1.55)	−0.54
Distance between cementum-enamel junction and free gingival margin, mm	1.35 (1.49)	1.62 (1.49)	−0.15	1.42 (1.50)	1.62 (1.49)	−0.11
Bleeding on probing, % sites per person	13.1 (22.3)	20.7 (27.4)	−0.26	12.7 (21.7)	20.7 (27.4)	−0.28

Data are mean (SD). The periodontal pocket depth measurements were made at three sites on the vestibular and lingual aspects of each tooth and the periodontal probing depth (PPD) score was calculated as a mean value divided by the number of sites examined. The Bleeding on Probing (BOP) index evaluated the buccal, lingual, mesial and distal sulci of all teeth based on the tendency to bleed after a standard stimulus. The four surfaces of each tooth were tested to provide a maximum total of 128 sites and the index is the percentage of sites positive for bleeding on probing for each participant. The Clinical Attachment loss score was calculated as the sum of the mean PPD (sum of all values divided by the number of sites examined (6 per tooth)) and the mean (free gingival margin [GM]-cementum-enamel junction [CEJ]) (sum of all values divided by the number of sites examined (2 per tooth)). ^a^Standardized differences of 0.2, 0.5 and 0.8 can be considered to represent small, medium and large differences, respectively [29]. These differences do not denote statistical significance

From the final cohort, all 1355 participants who had moderate to severe periodontitis were matched 1-to-1 on their propensity score for periodontal disease with 1355 participants who had no or mild periodontitis. The balance of clinical and socio-demographic characteristics, such as age, sex, race, time treated with dialysis, and comorbidity at baseline, was improved after matching on propensity score (Table 1) while characteristics denoting periodontitis remained appropriately separated (standardized differences >0.20) (Table 2).

**Fig. 2:**
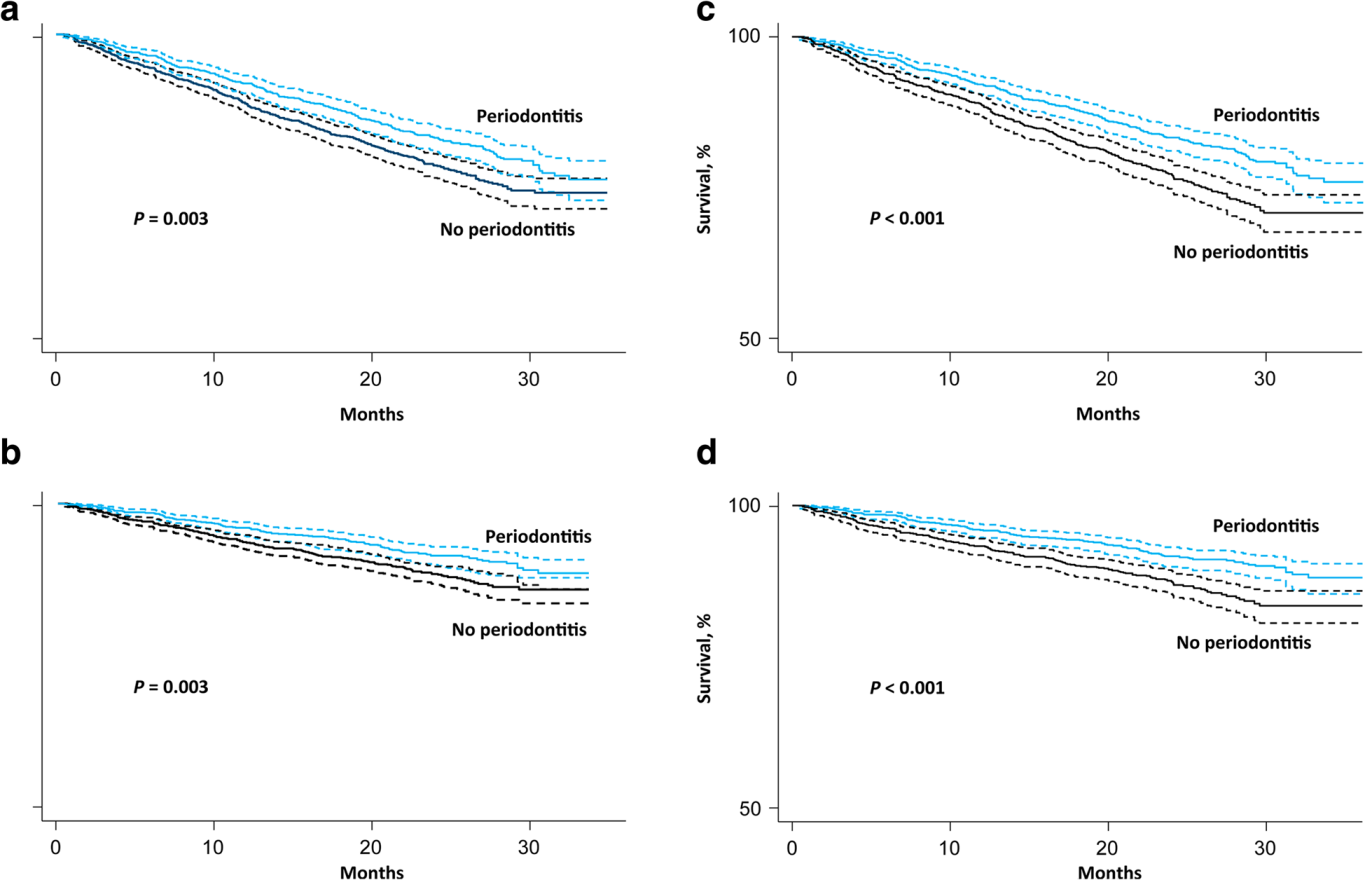
Kaplan-Meier survival plots (time until all-cause or cardiovascular death) for periodontitis in unmatched (*left side*) and matched (*right side*) samples. **a** All-cause mortality, unadjusted. **b** Cardiovascular mortality, unadjusted. **c** All-cause mortality, propensity-matched. **d** Cardiovascular mortality, propensity-matched

During a mean follow-up of 22.1 months (6150 person-years), 650 dentate participants died from any cause and 325 died due to a cardiovascular event. The cumulative incidence of any death was 91.2 per 1000 person-years in participants with moderate to severe periodontitis and 116.6 per 1000 person-years in participants with no or mild periodontitis in the overall cohort. The cumulative incidence of cardiovascular death was 42.6 per 1000 person-years in those with moderate to severe periodontitis and 60.6 per 1000 person-years in participants with no or mild periodontitis. In our unmatched cohort, overall survival and cardiovascular specific survival was longer among patients with periodontitis than in those without periodontitis for both the entire cohort and in the matched groups (Fig. 2).

When we used Cox proportional hazards models accounting for within-country clustering, the adjusted risk of all-cause mortality (hazard ratio (HR) 0.94 [95% confidence interval, (CI) 0.73 to 1.21]) and cardiovascular death (HR 0.91 [CI 0.64 to 1.29]) did not differ significantly between the participants based on the presence of periodontitis (Table 3). However, when analyses weighted participants according to their propensity score for periodontitis, those with moderate to severe periodontitis experienced a lower hazard of death from any cause (HR 0.83 [CI 0.68 to 1.00]) and cardiovascular death (HR 0.76 [CI 0.58 to 1.00]). Similarly, among matched participants, the risks of all-cause and cardiovascular death were lower in those with moderate to severe periodontitis (all-cause mortality, HR 0.74 [CI 0.61 to 0.90]; cardiovascular mortality HR 0.67 [CI 0.51 to 0.88]).

**Table 3:** Mortality outcomes with moderate to severe periodontitis among the whole cohort, propensity-weighted analyses and propensity-score matched patients with end-stage kidney disease treated with hemodialysis

		No (events per 100 person years)		
	Number of participants included in analyses	Moderate to severe periodontitis	No or mild periodontitis	Hazard ratio (95% CI)*
All-cause mortality				
Whole cohort	3338	242 (9.1)	408 (11.7)	0.94 (0.73 to 1.21)
Propensity-weighted	3338	242 (9.1)	408 (11.7)	0.83 (0.68 to 1.00)
Propensity-matched	2710	242 (9.1)	314 (13.0)	0.74 (0.61 to 0.90)
Cardiovascular mortality				
Whole cohort	3338	113 (4.3)	212 (6.1)	0.91 (0.64 to 1.29)
Propensity-weighted	3338	113 (4.3)	212 (6.1)	0.76 (0.58 to 1.00)
Propensity-matched	2710	113 (4.3)	167 (6.9)	0.67 (0.51 to 0.88)

Hazard ratios are reported for moderate to severe periodontitis (no or mild periodontitis is the reference group). CI denotes confidence interval. *Hazard ratios are controlled for age, sex, income, smoking, serum phosphorus, myocardial infarction, diabetes mellitus, mean arterial pressure, time on dialysis and number of teeth using a Cox proportional hazards regression model. Clustering by country was accounted for by random effects Cox proportional hazards regression fitted using a shared frailty model

### Sensitivity analyses

When we did additional analysis by propensity score methods matching patients with periodontitis which those without periodontitis and considering all variables included in the standard Cox proportional hazards regression model (*n* = 1078), the results were similar. The adjusted hazard ratio for all-cause mortality was 0.68 (CI 0.52 to 0.89) and cardiovascular mortality was 0.54 (CI 0.36 to 0.80).

We restricted analyses to those participants who had 12 natural teeth or more, as tooth loss may have indicated previous important periodontal disease and may have misclassified those with fewer teeth as having less severe periodontal disease. When the analyses were restricted to the 2229 participants who were dentate with 12 or more natural teeth, the results were similar although with wider confidence intervals (Additional file 2: Table S1).

When the cumulative incidence of cardiovascular death was calculated to account for other causes of death as competing risks, the results were comparable to main analyses, with a lower incidence of cardiovascular death for participants with moderate to severe periodontitis (cumulative incidence 14.9% with no or mild periodontitis compared with 10.5% with moderate or severe disease) (Additional file 2: Figure S1). Similar findings accounting for competing risks of death from non-cardiovascular causes were observed among matched participants (cumulative incidence of cardiovascular death 15.8% for participants without periodontitis versus 10.4% in participants with moderate to severe periodontitis when adjusted for non-cardiovascular causes of death).

## Discussion

ORAL-D is a large-scale prospective cohort study that was designed to examine oral diseases, including periodontitis, and associations with clinical outcomes in adults with kidney failure treated with long-term hemodialysis. Moderate to severe periodontitis was highly prevalent and affected about 40% of the cohort. Overall, there was no evidence of an association with all-cause or cardiovascular mortality in people treated with dialysis and who had moderate to severe periodontitis, when controlling for potentially confounding variables. In analyses in which participants were otherwise matched for key clinical and sociodemographic characteristics, those with moderate to severe periodontitis had a lower risk of all-cause and cardiovascular mortality, than those with no or mild periodontitis. Findings were similar when limited to adults with a minimum of 12 natural teeth and when competing causes for cardiovascular death were considered.

These findings may be in contrast to some studies in the general population and in adults with other chronic diseases, which show that periodontitis is associated with increased mortality and cardiovascular events [10, 31]. The study findings are consistent with other studies which show no association of periodontitis with mortality among patients with coronary artery disease and older adults [13, 32]. The lack of association between periodontitis and increased mortality among people with end-stage kidney disease has several potential explanations. First, the most likely explanation is that periodontitis may be associated with factors that are also linked to mortality, such as age, smoking, employment, and time treated with dialysis, but that the effects of these potential confounding variables linked to both periodontitis and mortality were reduced in these analyses through restriction to propensity-matched patients or statistical adjustment. Indeed, dental disease is linked to socioeconomic disadvantage, lower health literacy, rural dwelling, [33] cardiovascular risk, and reduced receptiveness to health advice and engagement with health services, [34] all of which are related to increased mortality [35–37]. It is possible these factors were minimized in this study by controlling for measured confounders that might be indicative of these broader determinants of both dental health and survival. Second, loss of tooth attachment in older adults caused by periodontal destruction due to long-term gingival inflammation might have led to misclassification of adults with milder periodontal pocket depth due to tooth attachment loss as having no or mild periodontitis when, in fact, shallow periodontal pocket depth may have been indicative of more advanced periodontal disease. While some prognostic studies have similarly classified periodontitis by considering gingivitis and periodontal pocket depth, and concluded periodontitis is linked to worse survival, [19] other studies have evaluated alveolar bone loss and tooth mortality as indicating of the natural evolution of severe periodontal loss [10]. Third, ORAL-D provided short-term outcome information for participants at 12–24 months after baseline, at which time the negative effect of periodontitis per se may not have been sufficiently relevant to risks of mortality.

Although observational studies in the non-dialysis setting have generally demonstrated that periodontitis is associated with poor health outcomes, a recent trial of nonsurgical periodontal treatment did not improve glycemic control [24]. A recent uncontrolled study in adults treated with peritoneal dialysis showed improved C-reactive protein and blood urea nitrogen levels following periodontal treatment, although the clinical relevance of the measured changes in these biochemical endpoints is uncertain [38]. In contrast, a randomized trial of periodontal treatment showed no evidence of sustained improvement in periodontal health or inflammatory markers at 6 months among 53 dialysis patients [25]. Ultimately, understanding any effects of periodontal treatment on survival in the setting of chronic kidney disease requires a large adequately-powered trial, although based on the findings of ORAL-D, a more convincing basis within observational studies is required before trials are planned.

The ORAL-D study has a number of strengths. First, the sample size of ORAL-D was sufficiently large to detect a meaningful difference in mortality adjusted for relevant socio-demographic and clinical variables and was geographically diverse, increasing the generalizability of the findings. Second, the oral examination was calibrated across study sites and valid methods for oral health assessment and periodontitis were used. WHO criteria in fact recommend CPI and a specially designed probe for the evaluation of the periodontal status [27]. Despite this may be not as sensitive as the Williams probe, mostly used in the routinely periodontal practice, the WHO probe was conceived for epidemiological studies. Because of the large ORAL-D study sample, CPI and not PISA score was used as validated epidemiological screening procedure in populations (at the time of recruitment, PISA score had been only used in one small size cohort study [32]). CPI remains till today the most used and validated method to evaluate severity and degree of periodontal disease. Third, participants showed important differences in key periodontal characteristics at baseline suggesting patients had differing exposures to periodontal pathology during the course of follow up.

However, potential limitations of ORAL-D should also be considered. Periodontal disease was measured after starting hemodialysis, long after any potential biological pathway between exposure and outcome was established. As with any cohort study, residual confounding is possible; in particular, ORAL-D did not measure variables linked to dental care access, oral health literacy, early life socioeconomic status, or other health behaviors that may have explained any linkages between periodontal disease and longer life expectancy. Because ORAL-D did not include patients with earlier stages of chronic kidney disease, the possibility that periodontitis is associated with mortality in adults with milder kidney disease cannot be ruled out. Although the oral examination was done using the World Health Organization methods and standardized and discussed across countries using a shared operational protocol, variation in the oral examination in each country was possible. We did not conduct formal testing of inter-examiner agreement between centers, which may have confounded the diagnosis of periodontal disease between countries.

## Conclusions

In conclusion, our data suggests that periodontitis is not associated with increased risks of all-cause and cardiovascular mortality in adults treated with hemodialysis.

## Additional files

Additional file 1: Oral disease in adults treated with hemodialysis: prevalence, predictors, and association with mortality and adverse cardiovascular events: the rationale and design of the ORAL Diseases in hemodialysis (ORAL-D) study, a prospective, multinational, longitudinal, observational, cohort study, Study protocol. (PDF 3348 kb)

Additional file 2: Table S1. Cox proportional hazards of mortality associated with periodontitis in adults with 12 or more teeth; **Figure S1**. Cumulative incidence of cardiovascular death by periodontitis severity after adjustment for competing causes of death, Appendix. (PDF 197 kb)

## Abbreviations

BOP: Bleeding on probing
CAL: Clinical attachment loss
CI: Confidence interval
CPI: Community periodontal index
HR: Hazard ratio
IPTW: Inverse probability of treatment weighting
ORAL-D: ORAL diseases in hemodialysis
PISA: Periodontal inflamed surface area
PPD: Periodontal pocket depth
SD: Standard deviation
WHO: World health organization
