# Supplementary material for: Periodontitis and early mortality among adults treated with hemodialysis: a multinational propensity-matched cohort study
Source: BMC Nephrol. 2017 May 22;18:166. doi: 10.1186/s12882-017-0574-x (PMC5440912; doi:10.1186/s12882-017-0574-x)

Appendix

Appendix Methods

The PPD was the distance between the free gingival margin and the apical extent of the periodontal pocket with three PPD measurements for each of the vestibular and lingual aspects of the tooth recorded and expressed as a mean score over all sites examined. The same probe was used to measure the distance between the cementum-enamel junction (CEJ) and the free gingival margin (GM). The CAL was calculated as the sum of these distances according to the standard formula CAL = PPD + (GM – CEJ). The BOP index evaluated the buccal, lingual, mesial and distal sulci of all teeth recommended by the World Health Organization based on the tendency to bleed after a standard stimulus and indicating periodontal inflammation. The four surfaces of each tooth were tested to provide a maximum total of 128 sites and the final index was the percentage of all examined sites positive for bleeding.

Appendix Table 1: Cox proportional hazards of mortality associated with periodontitis in adults with 12 or more teeth

		No (events per 100 person years)		
	**Number included in analyses**	**Moderate to severe periodontitis**	**No or mild periodontitis**	**Hazard ratio (95% CI)**
All-cause mortality				
Whole cohort	2229	149 (7.9)	193 (8.4)	0.98 (0.67 to 1.40)
Propensity-weighted	2229	149 (7.9)	193 (8.4)	0.78 (0.52 to 1.16)
Propensity-matched	1844	149 (7.9)	159 (9.7)	0.80 (0.61 to 1.04)
Cardiovascular mortality				
Whole cohort	2229	63 (3.3)	94 (4.1)	0.95 (0.57 to 1.60)
Propensity-weighted	2229	63 (3.3)	94 (4.1)	0.89 (0.51 to 1.56)
Propensity-matched	1844	63 (3.3)	82 (5.0)	0.66 (0.45 to 0.97)

Appendix Figure 1 Cumulative incidence of cardiovascular death by periodontitis severity after adjustment for competing causes of death

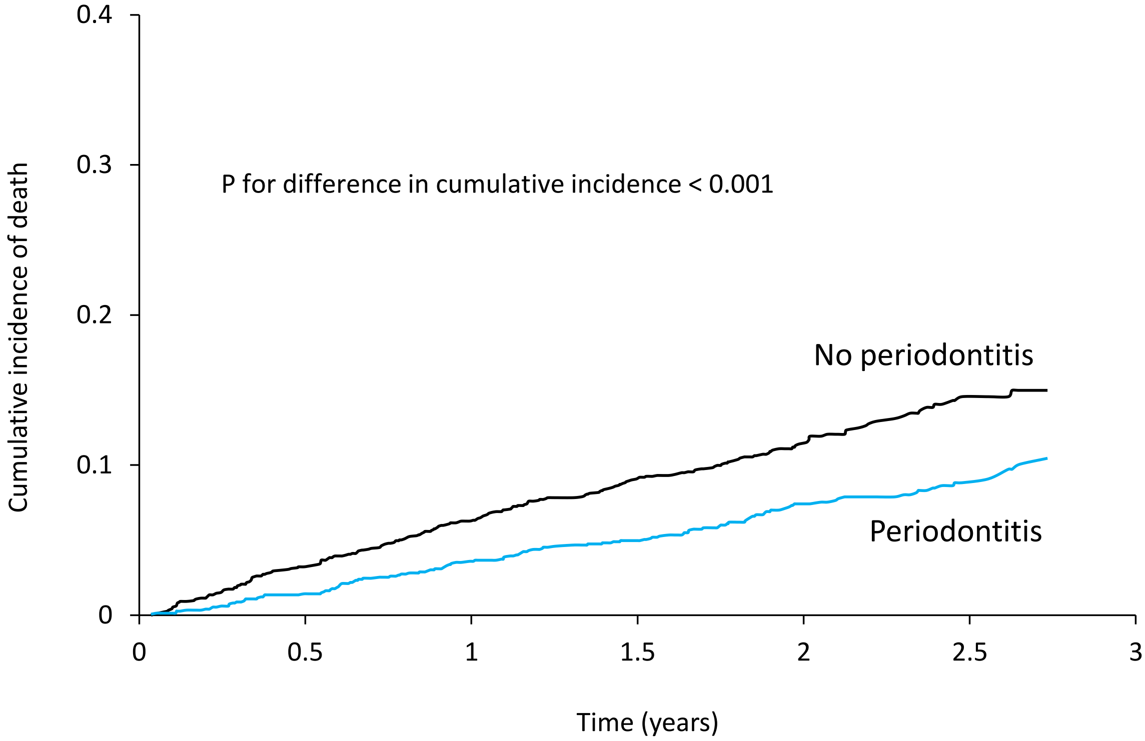

Supplement: Supplementary file 2 — Cox proportional hazards of mortality associated with periodontitis in adults with 12 or more teeth; Figure S1. Cumulative incidence of cardiovascular death by periodontitis severity after adjustment for competing causes of death, Appendix. (PDF 197 kb) [file 12882_2017_574_MOESM2_ESM.pdf]
